# Spatiotemporal Investigation of Antibiotic Resistance in the Urban Water Cycle Influenced by Environmental and Anthropogenic Activity

**DOI:** 10.1128/spectrum.02473-22

**Published:** 2022-08-29

**Authors:** Keira Tucker, Leonardos Mageiros, Alno Carstens, Ludwig Bröcker, Edward Archer, Katrin Smith, Evangelos Mourkas, Ben Pascoe, Daan Nel, Guillaume Meric, Samuel K. Sheppard, Barbara Kasprzyk-Hordern, Marelize Botes, Edward J. Feil, Gideon Wolfaardt

**Affiliations:** a Department of Microbiology, Faculty of Science, University of Stellenbosch, Matieland, Stellenbosch, South Africa; b Milner Centre for Evolution, Department of Biology and Biochemistry, University of Bath, Claverton Down, United Kingdom; c Department of Statistics and Actuarial Science, Stellenbosch University, Stellenbosch, South Africa; d Cambridge-Baker Systems Genomics Initiative, Baker Heart & Diabetes Institute, Melbourne, Victoria, Australia; e Department of Infectious Diseases, Central Clinical School, Monash University, Melbourne, Victoria, Australia; f Department of Chemistry, University of Bath, Bath, United Kingdom; g Department of Chemistry and Biology, Ryerson University, Toronto, Ontario, Canada; Connecticut Agricultural Experiment Station

**Keywords:** antimicrobial resistance, metagenomics, one health, wastewater, surface water, wastewater treatment

## Abstract

With increasing emergence of antimicrobial resistant bacteria (ARB) and the risk this poses to public health, there are growing concerns regarding water pollution contributing to the spread of antimicrobial resistance (AMR) through inadequate amenities and the rapid rate of urbanization. In this study, the impact of different anthropogenic factors on the prevalence of AMR in the urban water cycle in Stellenbosch, South Africa (SA) was examined. Carbapenem, colistin, gentamicin and sulfamethoxazole resistant Gram-negative bacteria were recovered by selectively culturing aqueous, biofilm and sediment samples from sites impacted to varying degrees by informal settlements, residential, industrial, and agricultural activities, as well as a municipal wastewater treatment works (WWTW). A metagenomic approach determined community profiles and dominant AMR genes at various sites, while carbapenem resistant colonies were characterized using whole genome sequencing (WGS). Isolates recovered from agricultural sites exhibited relatively high levels of resistance to carbapenems and colistin, whereas sites impacted by domestic run-off had a higher prevalence of resistance to gentamicin and sulfamethoxazole, corresponding to usage data in SA. Similar microbial taxa were identified in raw sewage, sites downstream of informal settlements, and industrial areas that have limited waste removal infrastructure while WWTW were seen to reduce the prevalence of ARB in treated wastewater when operating efficiently. The results indicate the multiple complex drivers underpinning environmental dissemination of AMR and suggest that WWTW assist in removing AMR from the environment, reinforcing the necessity of adequate waste removal infrastructure and antibiotic stewardship measures to mitigate AMR transmission.

**IMPORTANCE** The results from this study are of importance as they fill a gap in the data available on environmental AMR in South Africa to date. This study was done in parallel with co-investigators focusing on the prevalence of various antimicrobials at the same sites selected in our study, verifying that the sites that are influenced by informal settlements and WWTW influent had higher concentrations of antimicrobials and antimicrobial metabolites. The various locations of the sample sites selected, the frequency of the samples collected over a year, and the different types of samples collected at each site all contribute to informing how AMR in the environment might be affected by anthropogenic activity.

## INTRODUCTION

Antimicrobial resistance (AMR) is a global crisis predicted to cause 10 million annual deaths by 2050 (1). The ‘One Health’ approach (2) acknowledges the key role of animals and the environment in the emergence and spread of antibiotic resistant bacteria (ARB) and antibiotic resistance genes (ARGs). In particular, the role of environmental waters as a reservoir of AMR has gained increased recognition. While wastewater treatment works (WWTW) have been commonly considered as hot spots for the emergence of ARB and the source of AMR in the environment (3, 4), other components of the urban water cycle have received less attention. These include surface waters such as rivers, lakes or reservoirs used for human and animal consumption, industrial processes, and agricultural irrigation. In low- and middle-income countries (LMIC) such as South Africa (SA), WWTW are often poorly maintained and receive influent at, or above, operating capacity. As a result, the effluent may fall below regulatory standards leading to high levels of contamination in receiving waters (5). Furthermore, inadequate sanitation and related infrastructure in rapidly expanding informal settlements, often combined with poor maintenance of systems required for the delivery and collection of potable water, result in bypassing the treatment process altogether. The direct domestic use of untreated surface waters is therefore a pressing public health concern. In addition, used water contaminates the source water due to lack of wastewater removal infrastructure. Bucket toilets and open sewers are among the many unsanitary practices that occur in the informal settlements and contribute greatly to environmental pollution (6).

Increased use of antibiotics and development of resistance has led to a greater reliance on last resort antibiotics such carbapenems and colistin. As a result, resistance to these antibiotics has become a concern due to the limited treatment options available (7–9). In SA, 80% of clinically relevant antibiotics are also used for agricultural, livestock, and domestic animal purposes (10). These antibiotics are not only to treat infections, but also for preventative measures, and in the past have been used to facilitate growth promotion (11). As antibiotics for livestock are mostly added to animal feed, they have a direct effect on the animal’s gut microbiota. Therefore, the animals might act as a reservoir for resistant bacterial populations that can be excreted into the environment or be directly transmitted to humans (12, 13). Rates of AMR in South Africa have been increasing, particularly in the prevalence of multi-drug resistant tuberculosis (TB) and extended spectrum β-lactamases and carbapenem resistant *Enterobacteriaceae* (CRE) infections. Specifically, 80% of Acinetobacter. baumannii and 18% of Klebsiella. pneumoniae clinical infections in 2019 were resistant to carbapenems (14) Focusing on AMR in WWTW, may lead to a biased opinion that singles out WWTW as an important source and distributor of AMR in the urban hydrological cycle; however, WWTW receive their influent from various sectors of society that could ultimately be the source of ARB and antibiotic resistance genes (ARG). This study aimed to consider the various sectors of an urban setting that may contribute to AMR, and which potentially serve as hot spots, by determining resistance profiles from sites that are influenced by agriculture, industry, hospitals, as well as formal and informal residential dwellings that discharge sewage and greywater.

## RESULTS

### Percentage resistance.

**FIG 1:**
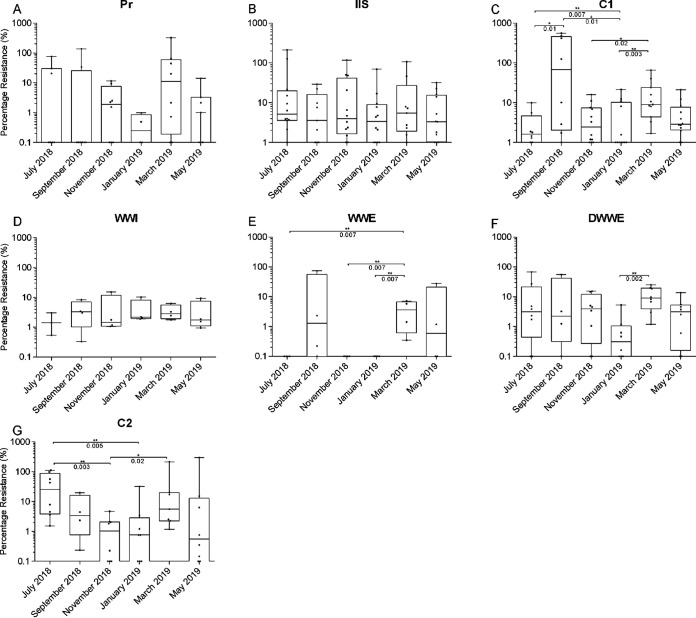
Percentage of culturable antibiotic resistant Gram-negative bacteria over time. The median percentage of antibiotic resistant bacteria is shown for each site at each sampling event. Pr - pristine site with minimal contamination, IIS - the combination of three sites located in industrial regions and downstream of an informal settlement, C1 - the convergence of clean and contaminated river streams, WWI – wastewater influent, WWE - wastewater effluent, DWWE - downstream of the wastewater effluent entry in the receiving river, C2 - convergence site of all the river streams. Individual data points are indicated by black dots and represent the percentage of resistant bacteria regardless of the matrix sampled or the antibiotic tested. Median values, interquartile ranges and maximum and minimum values of the data sets are indicated on the box plots. Statistical significance (*P* < 0.03) is indicated by the asterisks. The logarithmic scale has been used on the Y axis. As a result, values of 0 (where no resistance to a particular antibiotic was observed) are indicated as 0.01. Source data are provided in Supplementary Tables S6.1–S6.7.

For this study, 6 samples per site were collected over the course of 1 year, with resistance to 4 antibiotics in 3 different matrices being determined. The percentage of resistant bacteria was the most consistent throughout the year (no significant differences between months) at industrial and informal settlement (IIS) (Fig. 1B), followed by waste water influent (WWI) (Fig. 1D). These sites were significantly impacted by anthropogenic activity. While Pr, a site little affected by anthropogenic activity, also showed no significant differences between months (Fig. 1A), no resistance to antibiotics was observed for many of the samples. Greater temporal variability in the percentage of resistant bacteria between months were observed in sites that had less human activity in the surrounding areas (convergence of Plankenbrug and Eerste rivers [C1], downstream of wastewater effluent [DWWE]), and the convergence of all rivers (convergence of Eerste and Veldwagters rivers [C2]). There was a significant decrease (*P < *0.03) in the percentage of resistant bacteria in warmer months (November 2018 and/or January 2019) compared to cooler months (March-September), at C1, DWWE, and C2 (Fig. 1C, F, and G) where less influence from anthropogenic activity was evident. In addition, no antibiotic resistant bacteria were observed in the warmer months at wastewater effluents (WWE) and in July 2018 (Fig. 1E).

**FIG 2:**
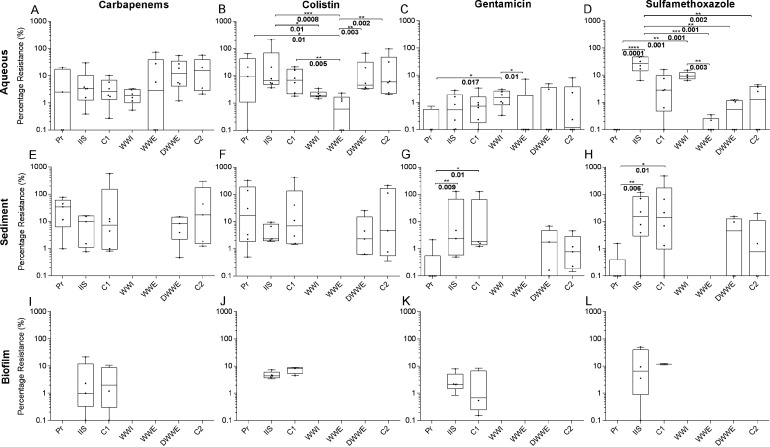
Percentage of culturable antibiotic resistant Gram-negative bacteria in various sample sites and matrices. The median percentage of Gram-negative bacteria that were resistant to carbapenems (A, E, I), colistin (B, F, J), gentamicin (C, G, K) and sulfamethoxazole (D, H, L) in aqueous, biofilm and sediment samples from all sampling points are shown. Pr - pristine site with minimal contamination, IIS - the combination of three sites located in industrial regions and downstream of an informal settlement, C1 - the convergence of clean and contaminated river streams, WWI – wastewater influent, WWE - wastewater effluent, DWWE - downstream of the wastewater effluent entry in the receiving river, C2 - convergence site of all the river streams. Individual data points are indicated by black dots and represent one of the six sampling events that occurred throughout the year. Median values, interquartile ranges and maximum and minimum values of the data sets are indicated on the box plots. Statistical significance (*P* < 0.03) is indicated by the asterisks. The logarithmic scale has been used on the Y axis. As a result, values of 0 (where no resistance to a particular antibiotic was observed) are indicated as 0.01. Source data are provided in Supplementary Tables S7.1–S7.4.

The median percentage of resistant bacteria for all matrices and sample sites ranged from 1–40% for carbapenems, 1–20% for colistin, 0–3% for gentamicin and 0–30% for sulfamethoxazole (Fig. 2). The median percentage of bacteria resistant to gentamicin across all sites were significantly lower than those resistant to carbapenems (*P < *0.05) and colistin (*P < *0.01), while a significantly higher percentage of colistin resistant bacteria was observed compared to sulfamethoxazole resistant bacteria (*P* < 0.05). Although statistically insignificant, the percentage of bacteria resistant to carbapenems in aqueous samples was slightly higher in sites situated further away from WWI (DWWE and C2). Similar percentages of resistance were observed in IIS - WWI for carbapenems (Fig. 2A). Bacteria in the aqueous samples from the WWTW (WWI and WWE) showed a significantly lower percentage resistance to colistin compared to the other sites (Fig. 2B) (*P < *0.03), with the difference between IIS and WWE being the most significant (*P = *0.0008). The opposite observation was made for gentamicin and sulfamethoxazole where the highest percentage of resistant bacteria were observed in WWI and IIS respectively, sites which are near human activity (Fig. 2C and D). Differences in the percentage of sulfamethoxazole resistant bacteria in aqueous samples were most significant between IIS and WWE, and IIS and Pr (Fig. 2D) (*p* < 0.001). The trends observed in the aqueous samples were also observed for the sediment samples for each antibiotic. The percentage of resistant bacteria at WWE for all antibiotics varied greatly over sampling events compared to WWI (Fig. 2A to D).

Although there was no overall significant difference in the percentage of culturable Gram-negative antibiotic resistant bacteria between the aqueous, sediment and biofilm samples (*P* > 0.05), a trend was apparent whereby resistance was most prevalent in sediment samples. For example, median resistance to gentamicin from the aqueous matrix at sites C2 and DWWE were 0.1% and 0% respectively, whereas the equivalent figures from the sediment samples at these sites were 0.8% and 2% (Fig. 2C and G). In biofilms, the highest median percentage resistance was seen for sulfamethoxazole (10%), followed by colistin, carbapenems and gentamicin. No large variation in the abundance of resistant bacteria between biofilm sites was observed, however resistance to colistin over the sampling period was consistent compared to the other antibiotics. The variation in percentage resistance in biofilm samples to colistin compared with the other antibiotics was minimal over the sampling duration. However, these results could be affected by the low number of biofilm samples that were collected.

### Whole community and single colony genomics.

**FIG 3:**
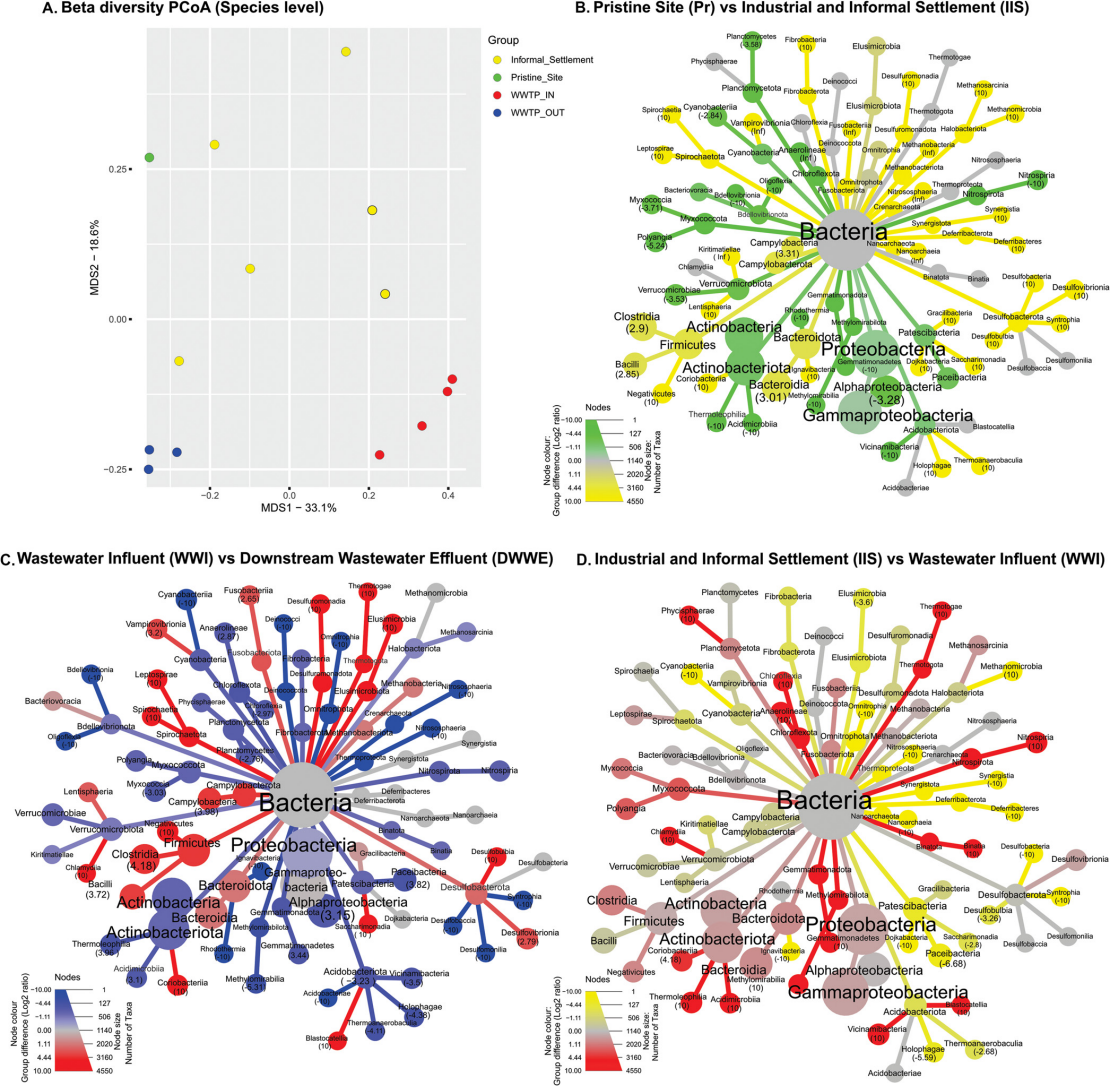
Diversity of whole community profiles in aqueous samples from selected sites. A. Beta diversity Principal Coordinates Analysis (PCoA) derived from the Shannon distances of microbial communities at the species level between Pr, IIS, WWI and DWWE. Principal Component 1 (PC1) and Principal Component 2 (PC2) explain 33.1% and 18.6% of the genetic diversity between samples respectively, for a total of 51.7% of the overall diversity. Source data are provided in Supplementary Table S8. Taxonomical comparisons are shown between B. Pr and IIS, C. WWI and DWWE and D. IIS and WWI. Taxa shown as colors were found to be abundant at the corresponding sampling site, while gray nodes indicate taxa that were found in similar proportions at each site. The depth of the color indicates the log_2_ ratio of median abundances between the two groups. Whenever the log_2_ ratio of a terminal node is larger than 2.5 or smaller than -2.5, its exact number appears within the parentheses below the taxon name to give a quantitative indication of how taxa are distributed between the sites. The tree is pruned down to the Class level for visualization purposes. The node size indicates the number of taxa included in the corresponding children nodes.

The beta diversity was found to vary between metagenomic samples (Fig. 3A). A Principal Coordinate Analysis (PCoA) showed that data points representing different sampling sites did not overlap, demonstrating that each sample site had different community compositions. Data points at WWI and DWWE were distinctly clustered suggesting that the communities from the cohort selected from these sites may be different from one another. This data also suggests that the community composition at these sites, particularly in DWWE, would be the most consistent over the course of the year, while the communities present at IIS would vary with time (Fig. 3A).

Dominant classes present in all metagenome sampling sites were *Gammaproteobacteria*, *Actinobacteria*, and *Alphaproteobacteria*. Taxa present at Pr were mostly dissimilar to taxa present in IIS, as indicated by the limited number of gray nodes in Fig. 3B. The 5 most abundant classes that are recovered from the WWTW are *Alphaproteobacteria*, *Clostridia*, *Bacteroidia*, *Gammaproteobacteria*, and *Actinobacteria* (Fig. 3C). When comparing the diversity in community composition between WWI and IIS (Fig. 3D), a higher proportion of the detected taxa were present in both sites (dull and/or gray nodes) in contrast to the other site comparisons (Fig. 3B and C). The similar abundances of some phyla at these 2 sites suggests that IIS has a microbial composition similar to that of untreated wastewater. Firmicutes, Bacteroidota, Actinobacteriota, and Proteobacteria are among the 4 most dominant phyla in the human gut (15), some of which are evident in both IIS and WWI samples. This suggests that IIS may contain untreated sewage as a result of the lack of sanitation infrastructure. In contrast, Fig. 3B and C indicate that the pristine site has a different community profile compared to water samples affected by human influence. A bar plot of the 10 most frequently observed classes in the whole community profiles can be seen in Fig. S1 while raw data for the ARGs detected in the samples are provided in Table S4.

For the single colony genomics, Pseudomonas spp. and Aeromonas spp. were the dominant genera in the carbapenem resistant isolates selected from the SuperCARBA plates and Pseudomonas spp. had the highest number of antibiotic resistance genes (Table 1). Carbapenem resistance genes were seen almost exclusively in Aeromonas spp. while peptide, aminoglycoside, and sulfonamide/trimethoprim resistance genes were observed over a wider variety of genera. Identification of these isolates to the species level is provided in Table S2, while raw data for the genes detected in each of these isolates are provided in Table S5. A number of the ARGs detected are core genes and may be responsible for intrinsic resistance to antibiotics.

**TABLE 1:** Number of unique target antibiotic resistance genes in each genus identified

Genus	No. of isolates	Carbapenems	Peptide	Aminoglycoside	Sulfonamide /trimethoprim	Multidrug (Core)^*a*^
Pseudomonas	61	1	3	15	9	71(6)
*Aeromonas*	40	6	4	15	7	55(2)
*Citrobacter*	6	0	2	13	4	48
*Chromobacterium*	5	0	0	0	3	1
*Stenotrophomonas*	4	0	2	6	4	57
Acinetobacter	2	0	0	0	1	11
*Comamonas*	2	0	0	0	0	0
*Chryseobacterium*	1	0	0	0	0	1
Enterobacter	1	1	1	3	0	24
*Hpunalikevirus*	1	0	0	2	0	13
Klebsiella	1	0	2	5	0	23
*Pandoraea*	1	0	0	0	0	3
*Pseudoxanthomonas*	1	0	0	0	0	4
*Raoultella*	1	0	1	3	2	21
*Shewanella*	1	0	0	0	0	0
Total unique genes		7	6	22	11	109

a Numbers outside the parentheses denote the total number of unique ARGs, of which the number within the parentheses is the number of core genes (the gene should be present in >90% of the isolates in the genera that contain >10 strains).

**FIG 4:**
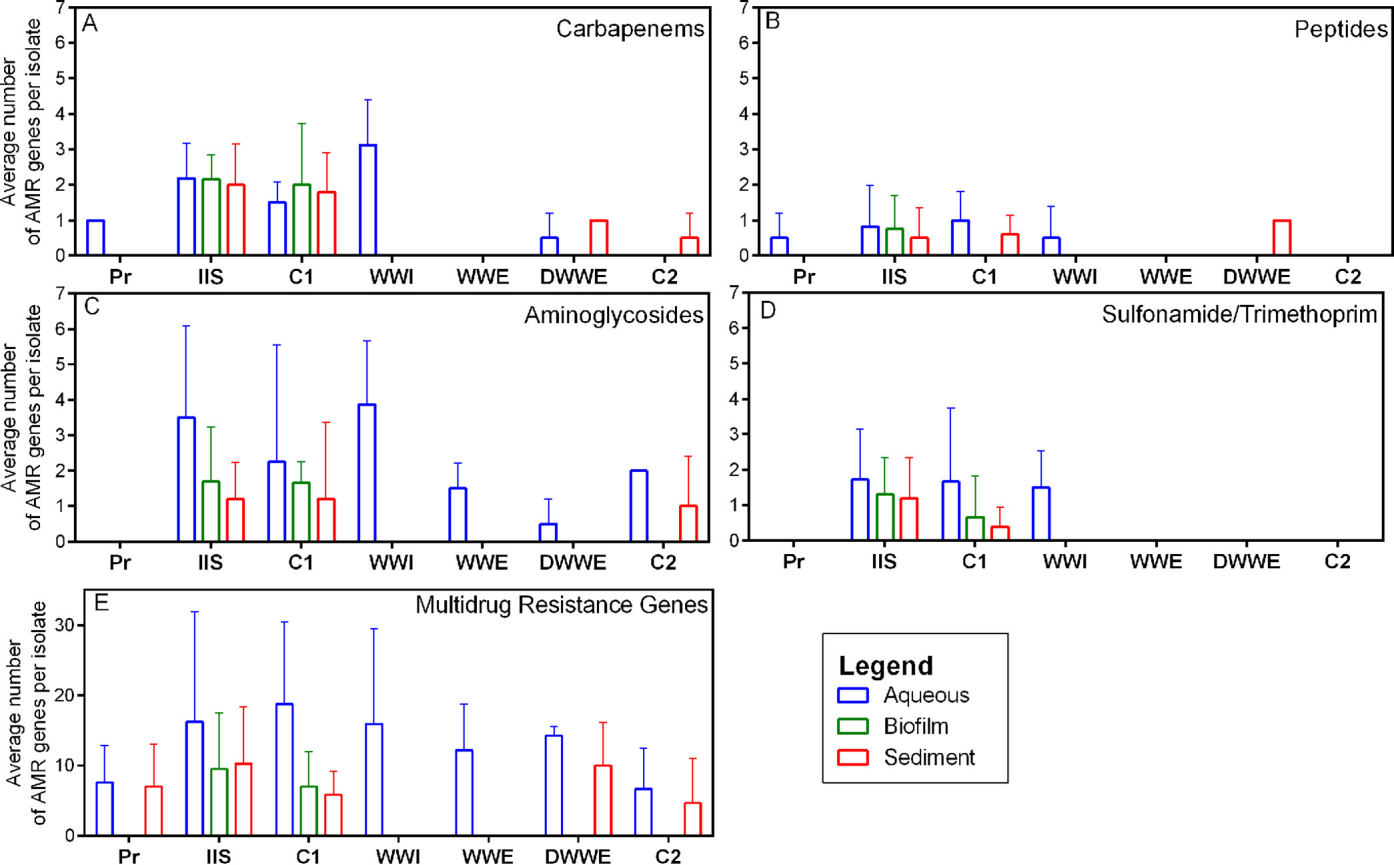
Average number of antibiotic resistance genes per isolate detected in single colonies. The average number of AMR genes conferring resistance to A. carbapenems, B. peptides, C. aminoglycosides, D. sulfonamides/trimethoprim, and E. multidrug resistance, in colonies isolated from aqueous (blue), biofilm (green) and sediment (red) samples on carbapenem media. Averages calculated were the sum total number of genes in the respective isolates from each sample type, divided by the total number of isolates obtained from the relevant sample. Source data are provided in Supplementary Tables S9.1–S9.5.

**FIG 5:**
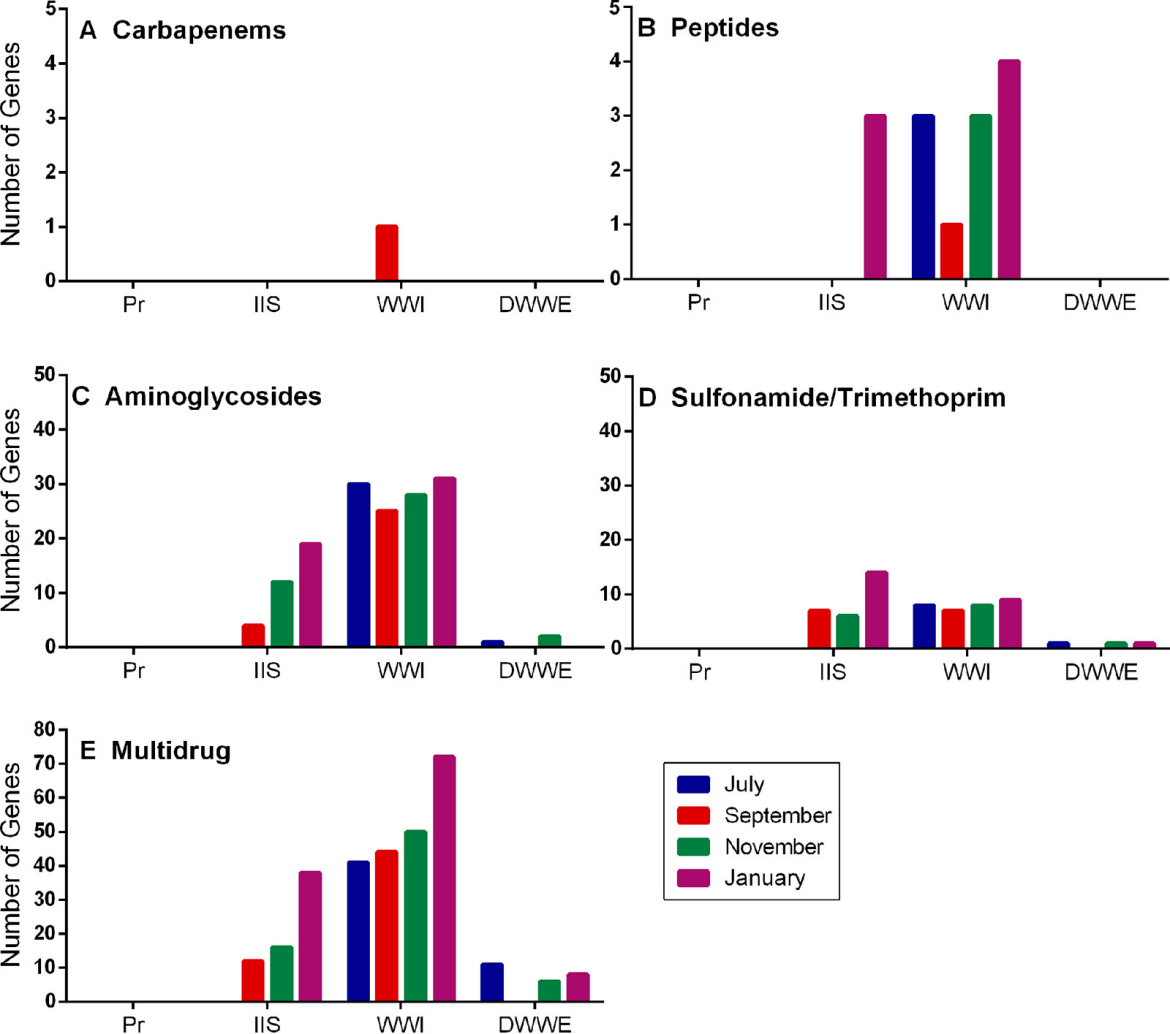
Number of target antibiotic resistance genes detected in metagenomic samples at each site. The number of target AMR genes detected in the whole community isolates present at selected sites, identified from whole community aqueous samples conferring resistance to A. carbapenems, B. peptides, C. aminoglycosides, D. sulfonamides/trimethoprim, and E. multidrug resistance detected in four different sampling events. Source data are provided in Supplementary Tables S10.1–S10.5.

Carbapenem resistant isolates obtained from IIS, C1, and WWI had a significantly higher average number of carbapenem resistance genes per isolate compared to the other sites further away from human activity (WWE, DWWE, C2; *P < *0.03) (Fig. 4A). These resistance genes were mainly observed in Aeromonas species, with the most prominent genes being *cphA5*, *cphA7*, and *imiH* (Table S5). The number of carbapenem resistance genes in the whole community was low, with only 1 (*ESP-1*) carbapenem specific resistance gene observed in WWI, and no carbapenem specific resistant genes in the whole communities at any of the other sites (Fig. 5A). While peptide specific resistance genes in single colonies were less prominent than carbapenem resistance genes, peptide resistance genes in the whole communities were observed at a higher abundance across months, with the majority being observed at WWI (Fig. 5B). Six different peptide resistance genes were found in carbapenem-resistant isolates, with the most prominent being *bacA*, *mdtM*, and *mcr-7.1* (Table S5). In contrast, 5 different peptide resistance genes were detected in the metagenomic samples, of which the most prominent was *ICR-Mo* (Table S4). Aminoglycoside resistance genes (conferring resistance to gentamicin) in single colonies were found at all sites except Pr and were the most prominent class of resistance genes targeting a single antibiotic across all sample sites (Fig. 4C). Of 22 aminoglycoside resistance genes detected in the carbapenem resistant isolates, *aadA*, *aph*(6)*-Id*, and *aph*(*3′'*)*-Ib* were the most common (Table S5). The number of antibiotic resistance genes was higher in the aqueous matrix compared to the biofilm and sediment samples at all sites. WWI had the highest average number of aminoglycoside resistance genes (4 genes per isolate for pure cultures isolated from WWI (Fig. 4C), and between 30 and 35 genes in whole communities (Fig. 5C), while WWE and DWWE (*P = *0.0032) had the least average number of resistance genes.

As trimethoprim is used in combination with sulfamethoxazole, genes conferring resistance to trimethoprim were included with sulfamethoxazole resistance genes. *Sul1*, *dfrA15*, and *dfrA14* were the most prominent genes in single colonies (Table S5), observed at IIS, C1, and WWI in numbers slightly higher than those found for peptides in individual isolates (Fig. 4B). S*ul1*, *sul2*, and *dfrA1* were the most abundant in whole communities (Table S4) and were mostly found at IIS and WWI (Fig. 5D).

While resistance genes specific to the target antibiotics selected in this study were detected in the sampling sites, numbers were relatively low compared to ARG that confer resistance to more than one class of antibiotic (multidrug ARG). 109 different multidrug ARG were detected in carbapenem resistant isolates, of which, *MexB*, *OpmH*, *and MexK* were the most prevalent, all of which were identified as core genes for Pseudomonas spp. (Table S5). Figure 4E shows that the highest number of these ARG were found in the aqueous samples at C1, where an average of 18.75 genes were found per isolate. At IIS and C1 where biofilm was collected, an average of 9.48 and 7 genes respectively per isolate were observed in this matrix. At the same sites, sediment was found to contain an average of 10.2 and 5.8 genes per isolate, respectively.

Similar to the single colonies, multidrug ARG were present in higher numbers compared to target ARG for all sites for whole communities (Fig. 5E). It was observed in whole communities that the number of target ARG in DWWE was reduced compared to WWI, similar to the reduced percentage of ARB found in WWE (Fig. 2B to D) (*P < *0.03). In contrast to the decrease in the percentage of resistant bacteria (Fig. 1) in the warmer months (November and January in SA), the number of resistance genes increased as the temperature increased (Fig. 5).

## DISCUSSION

**FIG 6:**
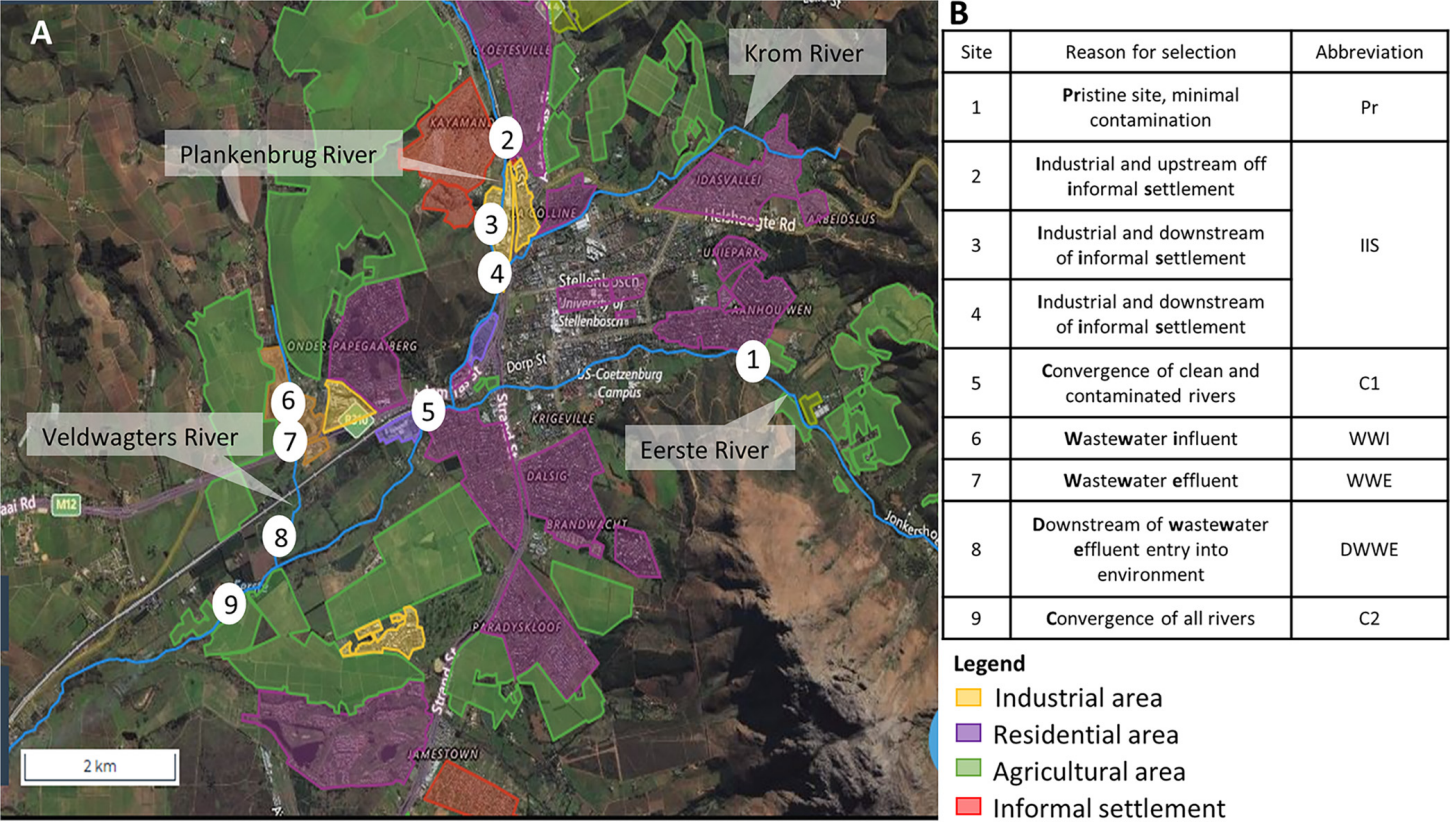
Map showing Stellenbosch rivers and sample sites. (A) A map indicating all the sampling sites and the surrounding areas of interest. Specifically the sites are: (1) Eerste river, before the influence of most sectors and used as a pristine site, (2) Plankenbrug river, upstream of informal settlement and industrial influence (3) Plankenbrug river, downstream of informal settlement and industrial influence (4) Plankenbrug river, downstream of informal settlement and industrial influence, (5) convergence of Eerste and Plankenbrug rivers, (6) wastewater treatment works influent, (7) wastewater treatment works effluent, (8) wastewater effluent enters Veldwagters river, (9) convergence of Eerste and Veldwagters rivers, (B). A table summarizing the sampling sites, the reason they were selected, and their abbreviation. For reporting purposes, sites 2, 3 and 4 are combined and referred to as “industrial and informal settlement” (IIS). The abbreviations indicated in the table will be used throughout the text.

Sulfamethoxazole, in combination with trimethoprim (co-trimoxazole), is used as a prophylactic antibiotic for HIV infected patients (16, 17). With 13.5% of the South African population living with HIV in 2019 (18), many use co-trimoxazole on a long-term basis. This could potentially lead to the emergence of co-trimoxazole resistant bacteria in informal settlements, as well as residential and hospital settings that enter the urban water cycle. Our results show some evidence of this as a significantly higher median percentage of bacteria resistant to sulfamethoxazole was found at sites in closer proximity to human populations and activities (sites 2 – 6 [IIS-WWI]) (Fig. 6) compared to the other sites that are influenced by agricultural activities, particularly Pr and DWWE (*P* < 0.001) (Fig. 2D). Previous studies have also shown that AMR is higher at locations with a greater human impact (19), with higher loads of antibiotics present in these water bodies as indicated by a study done in parallel to ours at the same sample sites (20). Development of resistance in these sectors may influence ARB evident at sites IIS, C1, and WWI through improper sanitation at IIS and C1, and an aggregation of waste from multiple sectors in the case of WWI. This is evident in Fig. 3D where the sites influenced by the informal settlement and industries including auto-mechanics, beverage manufacturing, butchers, and chemical manufacturing, had similar taxonomic profiles to WWI (Fig. 3D). It is acknowledged that the sample size for the community profiles at each site is limited, and differences and similarities in taxonomical profiles observed between sites may appear slightly different with a larger cohort, however the data presented in Fig. 3 still gives an indication of the differences between sites. As IIS was heavily polluted, these similarities, the similarities in the percentage of resistant bacteria at IIS and WWI (Fig. 2A to D), and the highly significant (*P* = 0.0008) reduction in bacterial resistance to some antibiotics from IIS to WWE (Fig. 2B), reinforces the need for adequate water treatment for all and preventing natural environmental surface waters having similar community profiles to untreated wastewater. While many suggest that WWTW are hot spots for environmental AMR dissemination (3, 4, 21, 22), our data indicates that WWTW assists in the removal of ARB and while they might be a reservoir for AMR in the treatment plant itself, through the treatment process, ARB are removed and therefore the WWTW is not a hot spot for AMR dissemination in the environment in this setting. We do however acknowledge that solids, such as those removed at the grit screens in the influent of the WWTW, as well as dewatered sludge, may harbor ARB that could enter the environment via alternative avenues if left untreated, however with applications such as thermal hydrolysis or anaerobic digestion for repurposing sludge, an unfavorable environment for ARB to persist would result (23).

The observed percentage of bacteria resistant to gentamicin is lower than expected, given its use for a number of clinical conditions (24) (Fig. 2C). The relatively high abundance of aminoglycoside resistance genes in both the carbapenem resistant isolates and in whole communities compared to other target antibiotic resistance genes (Fig. 4C and Fig. 5C) suggests that our estimates for the percentage of bacteria resistant to gentamicin are likely to be conservative, possibly due to the high concentration (32 μg/mL) of gentamicin added to the media. Many aminoglycoside resistance genes, including *aph(3′')-Ib*, *aph(*6*)-Id*, and *aadA*, have previously been detected in environmental and clinical isolates (25). These genes, as well as *sul1*, *mcr*, and *cph*, were mostly found in the single colony WGS data from anthropogenically impacted sample sites (Table S5), as well as the whole community profiling (Table S4). This suggests that these ARG may originate, or be selected, from human populations and are transferred to environmental waters through bacteria in sewerage, solid waste, and interaction with animals.

Since colistin is not extensively used in clinical settings and has been banned for use in livestock and veterinary purposes (26), the prevalence of colistin resistance at all sites where samples were collected was unexpected (Fig. 2B, F, and J). However, while there are rules surrounding the prescription of colistin in clinical cases, there are limited control measures in place in South Africa (27). Agricultural and aquaculture practices often don’t need veterinary antibiotic prescription and thus makes determining if the use of colistin in these settings is indeed halted a challenge (28). In addition, resistance due to extensive previous use in livestock could persist and disseminate, leading to the results observed at Pr, DWWE and C2, which have agricultural surroundings (Fig. 6) (29, 30). A similar scenario applies to carbapenems that are also not readily used in animals, but resistant organisms have been isolated from them nonetheless (31) and increased usage of carbapenems as a result of the increase of extended β-lactamase producing organisms has increased resistance to carbapenems (32). Rain and surface water runoff from these locations could lead to resistant organisms entering river waters. The most prominent plasmid mediated colistin resistance gene in the single colony data was mcr-7.1 (Table S5), which has previously been detected in high abundance from a wide range of environmental samples, including a zoo (33). *Mcr-7* has also been found in K. pneumoniae isolates obtained from chickens (34), while many other mcr genes have been identified in clinical isolates worldwide (35, 36).

When comparing the bacterial communities between DWWE and WWI, a larger variety of taxa were seen in DWWE (Fig. 3C). This is relevant to AMR, as there is a view that decreased species diversity in the environment allows ARB to disseminate more readily (37, 38). However, our data shows that colistin and carbapenem resistance at DWWE exceeded the abundance of resistant bacteria in WWI, while the opposite trend was seen for gentamicin and sulfamethoxazole (Fig. 2A to D). With the median levels of AMR remaining relatively constant across all sampling sites for carbapenems and colistin, intrinsic resistance to these antibiotics may be evident (39). Notably, colistin resistant isolates have been found in humans and animals that had no previous colistin treatment history, suggesting that resistance may occur without the presence of a selective pressure and maintenance of plasmids containing specific resistance genes (39). Likewise, carbapenem resistant *Enterobacteriaceae* (CRE) have been found to be prominent in both livestock and wildlife tissue and fecal samples (40, 41).

Efflux pumps function to export toxic or otherwise damaging compounds (including, but not limited to, antibiotics) out of the cell into the external environment. Genes encoding these pumps are often present on the chromosome, resulting in intrinsic resistance to multiple antibiotics even if this is not their primary function (42). This is evident in our isolates due to the presence of some genes encoding efflux pumps being core genes (Table S5). When exposed to low sub-inhibitory antibiotic concentrations, bacterial cells can upregulate the expression of efflux pumps, thus preparing the cell for exposure to higher concentrations that would otherwise be fatal (43). As a result, multidrug resistance genes present in environmental samples may play a greater role in conferring antibiotic resistance in Gram-negative bacteria in the environment compared to target-specific resistance genes. ARG were generally found to be reduced in the environment downstream from the treated wastewater discharge.

The majority of the single colony isolates were Pseudomonas spp. and Aeromonas spp. (Table 1) which is consistent with the metagenomics data showing that Gammaproteobacteria are the most prominent class across all sites (Fig. 3B to D). While Pseudomonas spp. and Aeromonas spp. are predominantly found in water environments (44–46), the selective approach to isolation may be inflating their prevalence further. As shown in Table 1, many of the isolates had multidrug efflux pumps, some of which were core genes which may have caused reduced susceptibility of the of these organisms to the selected antibiotic. However, predicting phenotypic resistance from genotype data can be difficult owing to changes in gene expression or the presence of unknown resistance mechanisms (47).

There is extensive temporal variation in the presence of resistant bacteria in WWE (Fig. 1E), compared to more consistent values in WWI (Fig. 1D). SA experiences scheduled power cuts known as load shedding, which distributes electricity consumption to prevent collapse of the power grid, in which a marked increase in resistant bacteria were observed in the effluent of March and May 2019, when load shedding was implemented (Fig. 1). Although generators are used in wastewater treatment plants, the delay in the switch from one power source to another may be a contributing factor in reducing the treatment efficiency and leading to the results observed.

Other factors affecting the treatment efficiency of WWTW and preventing the removal of ARB in WWE as seen in September 2018, March 2019, and May 2019 (Fig. 1E) could include population shifts during holiday periods in residential areas that have access to waste removal, seasonal changes that result in higher or lower rainfall, which in turn alters flow rates (48), and sporadic pollutants that enter the wastewater system. While some of these factors are not exclusive to WWTW and would apply to IIS as well, the sporadic anthropogenic activity at these sites (illegal dumping of refuse and waste), and the varied population, and socio-economic dynamics compared to those in suburban residential areas, leads to more consistent levels of AMR throughout the year (Fig. 1B).

In addition, the distance between the IIS data points in the PCoA plot (Fig. 3A) indicates that the informal settlement and industrial activity has an impact on microbial communities at different time points. Daily human activities, pollution, lack of sanitation and correct waste removal infrastructure described by Harvey (2018) may lead to shifting the proportion of ARB within these microbial communities in the environment seen in Fig. 2A to D which raises concern in settings where water treatment and distribution does not occur. The prevalence of ARG were lower at DWWE compared to WWI (*P* < 0.03 for aminoglycosides, carbapenems, and sulfamethoxazole) and other anthropogenically influenced sites (IIS; *P* < 0.03 for sulfonamides and carbapenems, and C1), suggesting that WWTW assisted in preventing ARB dissemination into the downstream environment (Fig. 4 and Fig. 5). As the microbial communities at WWI and DWWE were more clustered (Fig. 3A), and WWTW utilize microbes in some aspects of biological treatment processes (49), treatment facilities may play a role in the uniformity of the microbial communities that do enter the environment after passing through the WWTW and reiterates the importance of WWTWs in reducing AMR in the environment.

While individual studies on AMR in aqueous, sediment and biofilm samples have been performed previously, comparative studies between different matrices are limited. In general, the percentage of resistant bacteria in the sediment tended to be higher compared to aqueous or biofilm matrices (Fig. 2).

Biofilms are known to have an increased tolerance to antibiotics compared to planktonic as antibiotics may not be able to penetrate the biofilm due to the extracellular polymeric substance (EPS) acting as a diffusion-dominated barrier, meaning that susceptible cells at the base layer of the biofilm are protected from antibiotic exposure (47, 50–52). By homogenizing the biofilm for plating, these susceptible cells were exposed to antibiotics *in vitro*, meaning that our experimental set up did not closely mimic the natural environment. In future studies, using devices physically placed at the sample site to allow biofilms to attach and mature before being taken to the lab for analysis without disruption may give a better indication of AMR in environmental biofilms.

Aqueous samples, consisting of mostly planktonic cells that do not have EPS are more likely to contain ARB and ARG due to constant influx into river systems and various physiochemical properties that are constantly changing. In addition, shear forces from bulk-liquid allow biofilm erosion, where single or small groups of cells on the biofilm periphery are continuously detached from the matrix, forming part of the aqueous phase before attaching to a new surface (51). This may have been a contributing factor to the results observed where in some cases the median percentage resistance (Fig. 2), and the number of ARG in carbapenem resistant isolates (Fig. 4) were higher in the aqueous phase than in the biofilm and sediment.

### Conclusions.

Carbapenem and colistin resistance was found to be more prominent at sites influenced by agriculture compared to gentamicin and sulfamethoxazole, where resistance was found to be more prominent in sites closer to anthropogenic activity. Single colony ARB and ARG data was found to be representative of whole community metagenomics. Pseudomonas spp. and Aeromonas spp. were the most dominant genera with *cph*, *mcr-7.1*, *bacA*, *Aph 3′’Ib*, *Aph6-Id*, *aadA*, and *sul1* among the most prominent ARG, however genes encoding multidrug efflux pumps are suggested to play a larger role in environmental antibiotic resistance than specific plasmid mediated ARG. Metagenomic analysis showed similar taxa were observed in both IIS and WWI while taxa varied more when comparing WWI to DWWE. In addition, our results show that wastewater treatment removed bacteria resistant to most of the antibiotics tested in this study, suggesting that WWTW limit the dissemination of environmental AMR rather than being hot spots for AMR dissemination and are facilities that could aid in the removal of ARB. Sites affected by human activity showed the median levels of ARB were consistent throughout the year compared to sites with limited anthropogenic influence. While environmental AMR has been investigated previously, information based on a spatiotemporal approach of multiple sectors of society and matrices is limited. The data discussed in this study shows the complexity involved when trying to understand the impact of AMR in the environment. Ensuring access to water treatment infrastructure and waste removal, improving management and operation of these facilities, and changing behaviors may all play a role in mitigating AMR dissemination in the environment.

## MATERIALS AND METHODS

### Sampling sites and collection.

The Stellenbosch region near Cape Town, SA was selected as the site for this study due to the proximity of various societal sectors. Informal settlements, winelands, and agriculture including livestock farms, aquaculture, woodlands, and open land, as well as formal residential and industrial sectors are located within a 7 km radius of the town center. Aqueous, sediment and biofilm samples were collected from 10 sampling sites representing these diverse pressures indicated in Fig. 6 every other month for a period of 10 months (July 2018 - May 2019). For reporting purposes, the sites indicated in Fig. 6 were abbreviated as follows; pristine site with minimal contamination (Pr), the combination of three sites located in industrial regions and downstream of an IIS, the convergence of clean and contaminated river streams (C1), post-grit screen influent of a WWTW that serves a population of approximately 178 000, and receives mostly residential waste (WWI), and post UV treatment effluent of the WWTW (WWE) from a membrane bioreactor WWTW that uses UV disinfection in tertiary treatment opposed to chlorination, downstream of the wastewater effluent entry in the receiving river (DWWE), and a second convergence site of all the river streams (C2). For the remainder of this manuscript, the various sampling site locations will be referred to by the allocated abbreviations (Fig. 6).

Two grab aqueous samples (one 250 mL sample from all sampling sites, and the other, 1L from the Pr site, IIS sites, WWI and DWWE were collected in sterile Schott bottles from the mid-point between the surface and the bottom of the river. Biofilm samples were scraped from various parts of the under-side of rocks in the river using a toothbrush rinsed with 70% ethanol. The collected samples were then placed in sterile 50 mL falcon tubes containing 15 mL phosphate-buffered saline (PBS). Collection of sufficient biofilm material was possible at IIS and the convergence of the clean and contaminated rivers (C1). Sediment samples were collected from all sites, except at WWI and WWE. These samples were collected from the bottom of the riverbed or at the lowest depth possible in 15 mL falcon tubes. Due to the flow rate of the rivers and WWTW, and the way the biofilm and sediment samples were collected, they were considered heterogeneous and therefore technical repeats were deemed sufficient for the purpose of this study. All samples were collected in the morning and transported on ice back to the laboratory where they were stored at 4°C until processing. All processing occurred within 6 h of returning to the lab.

### Enrichment of highly resistant Gram-negative bacteria.

Gram-negative bacteria were targeted for ARB enumeration in this study. Four antibiotics considered a priority for study by the World Health Organization were selected; 2 are last resort antibiotics (carbapenems and colistin), and 2 are frequently used in a South African setting (gentamicin and sulfamethoxazole).

Serial dilutions (1:10) from each 250 mL aqueous sample were made in PBS, and 100 μL of each dilution was spot plated in duplicate onto MacConkey agar (which served as positive control selecting for culturable Gram-negative bacteria present in the sample), MacConkey agar supplemented with 512 μg/mL sulfamethoxazole (GLS), 32 μg/mL gentamicin sulfate (Melford) and 8 μg/mL colistin sulfate (Sigma-Aldrich) to select for Gram-negative bacteria resistant to sulfamethoxazole, gentamicin and colistin, and mSuperCARBA agar (CHROMagar) to select for carbapenem resistant Gram-negative bacteria. Due to the increased use of carbapenems and the increase in CRE in clinical environments, mSuperCARBA agar was used due to its chromogenic selective properties that would allow us to select for presumptive species of relevance. Antibiotic concentrations used were higher than the Clinical and Laboratory Standards Institute and European Committee on Antimicrobial Susceptibility Testing resistance breakpoints (53, 54) to select for highly resistant organisms in the sample.

Each sediment and biofilm sample was distributed into 2 mL microfuge tubes and centrifuged at 9900 × *g* for 10 min. The supernatant was discarded, and 100 mg wet weight was added to 900 μL PBS to make a 1:10 dilution. Each sample was vortexed thoroughly, a dilution series was made, and spot plates were performed as above.

All plates were incubated at 37°C for 18–24 h and resulting colonies were enumerated. CFU/mL for aqueous, and CFU/g for sediment and biofilm were calculated. The percentage of culturable Gram-negative bacteria resistant to each antibiotic was calculated as follows:

$$\%\text{ resistance}=\frac{\mathrm{CFU}/\mathrm{ml}(g)\;\mathrm{on}\;\mathrm{antibiotic}\;\mathrm{agar}}{\mathrm{CFU}/\mathrm{ml}(g)\;\mathrm{on}\;\mathrm{MacConkey}\;\mathrm{agar}}\;\times 100$$

(adapted from the Antimicrobial Resistance Percentages Rate Table [55]).

We note that using this approach it is possible to exceed a maximum value of 100% as applying a selective pressure on the microbial communities during culturing has the potential to select for antibiotic resistant bacterial species that have a faster growth rate compared to antibiotic susceptible species that may predominate the sample and grow on MacConkey agar without antibiotic supplementation but have a slower growth rate or varied growth requirements. As a result, a lower number of bacteria growing on MacConkey agar compared to the numbers on antibiotic supplemented agar can be evident (56).

### Whole genome sequencing.

Single colonies from each site and matrix were picked from SuperCARBA agar plates previously enumerated. Species were presumed chromogenically from manufacturers recommendations: Escherichia. coli (pink), Pseudomonas aeruginosa (cream to green), A. baumannii (cream), Klebsiella spp. (blue). Each selected colony was re-streaked on SuperCARBA media until a pure culture was obtained and was subsequently inoculated into 5 mL Terrific Broth and incubated overnight at 37°C. Genomic DNA from each overnight culture was extracted (57). Complete protocol can be found in Text S1.

Extracted DNA from 132 isolates was sent to MicrobesNG (Birmingham, UK) where Illumina MiSeq sequencing (2 × 250 bp paired end reads) was conducted with in-house quality control (adapter trimming with Trimmomatic v0.30, with a sliding window quality score cutoff value of Q15). Genus and species identification was performed with Kraken2 2.0.8 (58) and MetaPhlAn2 2.96 (59). ARG relevant to the antibiotics chosen to select for antibiotic resistant bacteria in this study were identified for each isolate using the Comprehensive Antibiotic Resistance Database (CARD) (60) and Resistance Gene Identifier (RGI; 5.1.1). An antimicrobial resistance gene was considered present only if the corresponding reads covered > 80% of its length. If more than 10 isolates were identified as the same genus, individual ARGs that were detected in > 90% of those isolates were denoted as core genes for that genus. For reporting purposes, the number of unique genes detected in a genus is reported rather than the total number of genes across all isolates.

### Whole community analysis.

Acknowledging the bias that culture methods introduce, in order to ensure the data obtained from the WGS from the selected single colonies was indeed representative of the sample, metagenomic sequencing of the whole samples was also performed. Comparing the data would be able to inform if metagenomic sequencing was necessary for future studies or if WGS gives an accurate representation of the sample.

The 1L aqueous samples from the Pr site, IIS sites, WWI and DWWE were filtered through 0.22 μm mixed cellulose ester filters (MilliPore). The turbidity of the samples determined the volume filtered (Table S1). The filtrate was discarded, and the filter was placed in a petri dish containing 4 mL citrate buffer (0.3M, pH 3.5) for 3 min (61). Remaining solid matter was scraped off the filter using a sterile blade, and the filter was discarded. The solution was placed in microfuge tubes and centrifuged at 15000 × *g* for 2 min. The pellet was washed with PBS and spun again, after which it was resuspended in 200 μL PBS. Total DNA was extracted from prepared aqueous samples, using the Quick-DNA Fecal/Soil Microbe Miniprep Kit (Zymo Research) according to manufacturer’s specifications.

Extracted DNA from 14 metagenomic samples from 5 sampling sites in 4 different sampling periods (Table S1), was sent to MicrobesNG (Birmingham, UK) where Illumina HiSeq sequencing (rapid run, 2 × 250 bp paired end reads) was conducted with in-house quality control (adapter trimming with Trimmomatic v0.30, with a sliding window quality score cutoff value of Q15). Taxonomical profiling was performed with Kraken2 2.0.8 and Bracken 2.5 (62) using the Genome taxonomy database for improved performance (GTDB) (63). ARG relevant to the antibiotics chosen to select for antibiotic resistant bacteria in this study were identified using CARD and Resistance Gene Identifier (RGI; 5.1.1). An antimicrobial resistance gene was considered present only if the corresponding reads covered > 80% of its length. For additional information on the metagenomic and whole genome samples, including sample sites, dates, and volumes, see Table S1 and Table S2 respectively. An inhouse R script was used to process the metagenomic data. The session_info output is given in Text S2. Low abundance taxa were filtered using a pre-processing step by removing taxa that had non-zero values in ≤ 10% of samples. This step reduced the number of identified species from 9,552 to 4,549 (Table S3). Beta diversity between samples was calculated using Shannon Beta diversity metric.

### Statistical analysis.

Repeated measures ANOVA with the assumption of compound symmetry over months was used. Fisher’s Least Significant Difference (LSD) *post hoc* test was performed to detect significant differences between sites over time using Statistica Software (TIBCO Software Inc. [2019]). Data Science Workbench (v13) was used to compare dates, sites, matrices, and antibiotics. A *P* value threshold of 0.05 was used for significance. No statistical test was used in the metagenomic samples for the differences between species abundance of different sites due to the low number of samples. Instead, the Log_2_ ratio was calculated and plotted using the metacoder 0.3.4 package. Finally, statistical significance for the pairwise comparisons between the groups for Fig. 1, 2 and 4 was inferred using Dunn’s test in GraphPad Prism 7.04 using a *P* value threshold of 0.03 for significance.

### Data availability.

The data sets supporting the conclusions of this article are included within the article and its additional files. The raw reads of the sequences used in this project can be found in BioProject PRJNA837742. The script written to analyze the metagenomic data is available upon request. Source data files for generation of Figures are in Tables S6–S10.
