# Supplementary material for: Spatiotemporal Investigation of Antibiotic Resistance in the Urban Water Cycle Influenced by Environmental and Anthropogenic Activity
Source: Microbiol Spectr. 2022 Aug 29;10(5):e02473-22. doi: 10.1128/spectrum.02473-22 (PMC9603458; doi:10.1128/spectrum.02473-22)
Supplement: Supplemental file 1 — Supplemental material. Download spectrum.02473-22-s0001.pdf, PDF file, 0.9 MB [file spectrum.02473-22-s0001.pdf]

Supplementary Material

Spatiotemporal investigation of antibiotic resistance in the urban water cycle influenced by environmental and anthropogenic activity

Text S1: DNA extraction protocol adapted from Crouse and Amorese (1987) used for single colonies.

1. Harvest 2 ml of an overnight bacterial culture by centrifugation at 2470 x g for 3 minutes in 2 ml microfuge tubes. Wash once with sterile TE buffer (*be gentle and quick - some bacteria may tend to start lysing in TE*).

2. Resuspend cell pellets in 500 µl SET buffer (25% sucrose, 2mM EDTA, 50mM Tris pH8).

3. Add protease K to 1 mg/ml. Incubate at 37 °C for 30 minutes.

4. Split the cell suspension into 2 x 500 µl in two microfuge tubes and add 500 µl TE to each.

6. Lyse cells with 50 µl 10% SDS. *Mix gently- the solution should become clear and gloopy*.

7. Incubate at 50 °C overnight. *This is step allows the proteinase K to degrade protein debris*.

8. Add 500 µl 7.5 M ammonium acetate to each tube and mix well. Hold at room temperature for 60 minutes. *This step is used to precipitate protein from solution*.

9. Spin at 16 700 x g for 15 minutes at room temperature to precipitate proteins. Place 500 µl of the supernatant into clean 2 ml microfuge tubes and add 2 volumes of 100% ethanol.

10. Leave overnight at 4 °C.

11. Centrifuge at 16 700 x g for 30 minutes at room temperature.

12. Carefully remove the supernatant from the DNA pellet and wash the pellet with 70% ethanol. Centrifuge at 16 700 x g for 5 minutes. Remove the supernatant and allow pellet to dry completely.

13. Resuspend the DNA of one tube in 200 µl TE buffer and combine with the other sample tube. Add RNase (final concentration 100 ng/ml). Incubate at 37 °C for 30 minutes.

References

Crouse, J., & Amorese, D. (1987). Ethanol precipitation: ammonium acetate as an alternative to sodium acetate. Focus, 9(2), 3–5.

Table S1: Metadata for whole community genomics. Sequencing read information and metadata for metagenome samples from each site.

Sample name	Site	Sample Date	# Bases	# Bases - post trimming	# Reads	# Reads - post trimming	% duplicates	mean read length	median read length	mode read length	Matrix	Volume filtered (ml)	DNA concentration (ng/ul)
A2_6	WWI	18/7/2018	9,830,865,294	9,344,512,356	39,166,794	39,165,432	1.7	238.2	251	251	Aqueous	250	61.3
A2_8	DWWE	18/7/2018	56,499,901,710	42,406,539,347	225,099,210	225,086,800	1.2	187.2	251	251	Aqueous	1000	4.4
A3_2	IIS	17/9/2018	21,309,144,992	16,822,236,198	84,896,992	84,884,304	0.3	197.4	251	251	Aqueous	250	12.8
A3_3	IIS	17/9/2018	8,595,781,642	8,336,568,989	34,246,142	34,245,108	1.5	243.3	251	251	Aqueous	500	29.5
A3_6	WWI	17/9/2018	11,448,494,030	10,876,311,882	45,611,530	45,609,838	1.2	238.1	251	251	Aqueous	150	24.4
A4_1	Pr	17/11/2018	6,898,629,078	6,692,965,212	27,484,578	27,483,698	2.7	243.4	251	251	Aqueous	1000	13.4
A4_2	IIS	17/11/2018	6,806,437,782	6,678,658,197	27,117,282	27,116,052	1	246.2	251	251	Aqueous	500	71.8
A4_3	IIS	17/11/2018	7,944,049,098	7,740,793,752	31,649,598	31,648,334	1.8	244.4	251	251	Aqueous	350	4.8
A4_6	WWI	17/11/2018	7,615,196,428	6,132,658,820	30,339,428	30,337,396	0.2	200.8	251	251	Aqueous	150	94.3
A4_8	DWWE	17/11/2018	8,188,321,796	8,001,007,092	32,622,796	32,622,188	2.7	245.3	251	251	Aqueous	1000	26.3
A5_2	IIS	22/1/2019	7,854,620,810	7,487,452,636	31,293,310	31,292,144	1.3	239.1	251	251	Aqueous	150	31.3
A5_3	IIS	22/1/2019	8,332,827,014	7,757,431,031	33,198,514	33,197,304	1.1	233.1	251	251	Aqueous	210	26.5
A5_6	WWI	22/1/2019	8,369,642,690	8,148,419,949	33,345,190	33,344,562	1.5	244.1	251	251	Aqueous	100	26.9
A5_8	DWWE	22/1/2019	7,923,649,324	7,737,774,422	31,568,324	31,567,768	2.1	245.1	251	251	Aqueous	1000	10.6

Text S2: Identification of each isolate at each site, and sequencing read information.

R script session output information

R version 3.6.3 (2020-02-29)

Platform: x86_64-w64-mingw32/x64 (64-bit)

Running under: Windows 10 x64 (build 18363)

Matrix products: default

locale:

[1] LC_COLLATE=English_Australia.1252 LC_CTYPE=English_Australia.1252 LC_MONETARY=English_Australia.1252 LC_NUMERIC=C LC_TIME=English_Australia.1252

attached base packages:

[1] stats4 parallel stats graphics grDevices utils datasets methods base

other attached packages:

[1] data.table_1.13.6 hrbrthemes_0.8.0 devtools_2.3.2 usethis_2.0.0 ggrepel_0.9.1 metacoder_0.3.4

[7] taxa_0.3.4.9001 DESeq2_1.26.0 SummarizedExperiment_1.16.1 DelayedArray_0.12.3 BiocParallel_1.20.1 matrixStats_0.57.0

[13] GenomicRanges_1.38.0 GenomeInfoDb_1.22.1 IRanges_2.20.2 S4Vectors_0.24.4 vsn_3.54.0 Biobase_2.46.0

[19] BiocGenerics_0.32.0 apeglm_1.8.0 microbiome_1.8.0 phyloseq_1.30.0 gridExtra_2.3 rmarkdown_2.6

[25] edgeR_3.28.1 limma_3.42.2 ggpubr_0.4.0 dendextend_1.14.0 vegan_2.5-6 lattice_0.20-38

[31] permute_0.9-5 zCompositions_1.3.4 truncnorm_1.0-8 NADA_1.6-1.1 survival_3.1-8 MASS_7.3-53.1

[37] compositions_2.0-1 htmlwidgets_1.5.3 autoplotly_0.1.2 cluster_2.1.0 ggfortify_0.4.11 heatmaply_1.1.1

[43] plotly_4.9.3 viridis_0.5.1 viridisLite_0.3.0 RColorBrewer_1.1-2 reshape2_1.4.4 readxl_1.3.1

[49] forcats_0.5.0 stringr_1.4.0 dplyr_1.0.2 purrr_0.3.4 readr_1.4.0 tidyr_1.1.2

[55] tibble_3.0.4 ggplot2_3.3.3 tidyverse_1.3.0 broom_0.7.3

loaded via a namespace (and not attached):

[1] tidyselect_1.1.0 RSQLite_2.2.2 AnnotationDbi_1.48.0 grid_3.6.3 TSP_1.1-10 Rtsne_0.15 munsell_0.5.0

[8] codetools_0.2-16 preprocessCore_1.48.0 withr_2.3.0 colorspace_2.0-0 knitr_1.28 rstudioapi_0.13 robustbase_0.93-6

[15] bayesm_3.1-4 ggsignif_0.6.0 Rttf2pt1_1.3.8 bbmle_1.0.23.1 GenomeInfoDbData_1.2.2 bit64_4.0.5 rhdf5_2.30.1

[22] rprojroot_2.0.2 coda_0.19-4 vctrs_0.3.6 generics_0.1.0 xfun_0.15 R6_2.5.0 seriation_1.2-9

[29] locfit_1.5-9.4 bitops_1.0-6 assertthat_0.2.1 scales_1.1.1 nnet_7.3-12 gtable_0.3.0 affy_1.64.0

[36] processx_3.4.5 rlang_0.4.10 genefilter_1.68.0 systemfonts_0.3.2 splines_3.6.3 extrafontdb_1.0 rstatix_0.6.0

[43] lazyeval_0.2.2 checkmate_2.0.0 BiocManager_1.30.10 abind_1.4-5 modelr_0.1.8 backports_1.2.0 Hmisc_4.4-2

[50] extrafont_0.17 tensorA_0.36.2 tools_3.6.3 affyio_1.56.0 ellipsis_0.3.1 biomformat_1.14.0 sessioninfo_1.1.1

[57] Rcpp_1.0.5 plyr_1.8.6 base64enc_0.1-3 progress_1.2.2 zlibbioc_1.32.0 RCurl_1.98-1.2 ps_1.5.0

[64] prettyunits_1.1.1 rpart_4.1-15 haven_2.3.1 fs_1.5.0 magrittr_2.0.1 openxlsx_4.2.3 reprex_0.3.0

[71] mvtnorm_1.1-1 pkgload_1.1.0 xtable_1.8-4 hms_0.5.3 evaluate_0.14 XML_3.99-0.3 rio_0.5.16

[78] emdbook_1.3.12 jpeg_0.1-8.1 testthat_3.0.1 compiler_3.6.3 bdsmatrix_1.3-4 crayon_1.3.4 htmltools_0.5.0

[85] mgcv_1.8-31 Formula_1.2-4 geneplotter_1.64.0 lubridate_1.7.9.2 DBI_1.1.0 dbplyr_2.0.0 Matrix_1.2-18

[92] ade4_1.7-16 car_3.0-10 cli_2.2.0 igraph_1.2.5 pkgconfig_2.0.3 registry_0.5-1 numDeriv_2016.8-1.1

[99] foreign_0.8-75 xml2_1.3.2 foreach_1.5.1 annotate_1.64.0 multtest_2.42.0 webshot_0.5.2 XVector_0.26.0

[106] rvest_0.3.6 callr_3.5.1 digest_0.6.27 Biostrings_2.54.0 cellranger_1.1.0 htmlTable_2.1.0 gdtools_0.2.3

[113] curl_4.3 lifecycle_0.2.0 nlme_3.1-144 jsonlite_1.7.2 Rhdf5lib_1.8.0 carData_3.0-4 desc_1.2.0

[120] fansi_0.4.1 pillar_1.4.7 pkgbuild_1.2.0 httr_1.4.2 DEoptimR_1.0-8
remotes_2.2.0 glue_1.4.2

[127] zip_2.1.1 png_0.1-7 iterators_1.0.13 bit_4.0.4 stringi_1.5.3
blob_1.2.1 memoise_1.1.0

[134] latticeExtra_0.6-29 ape_5.4-1

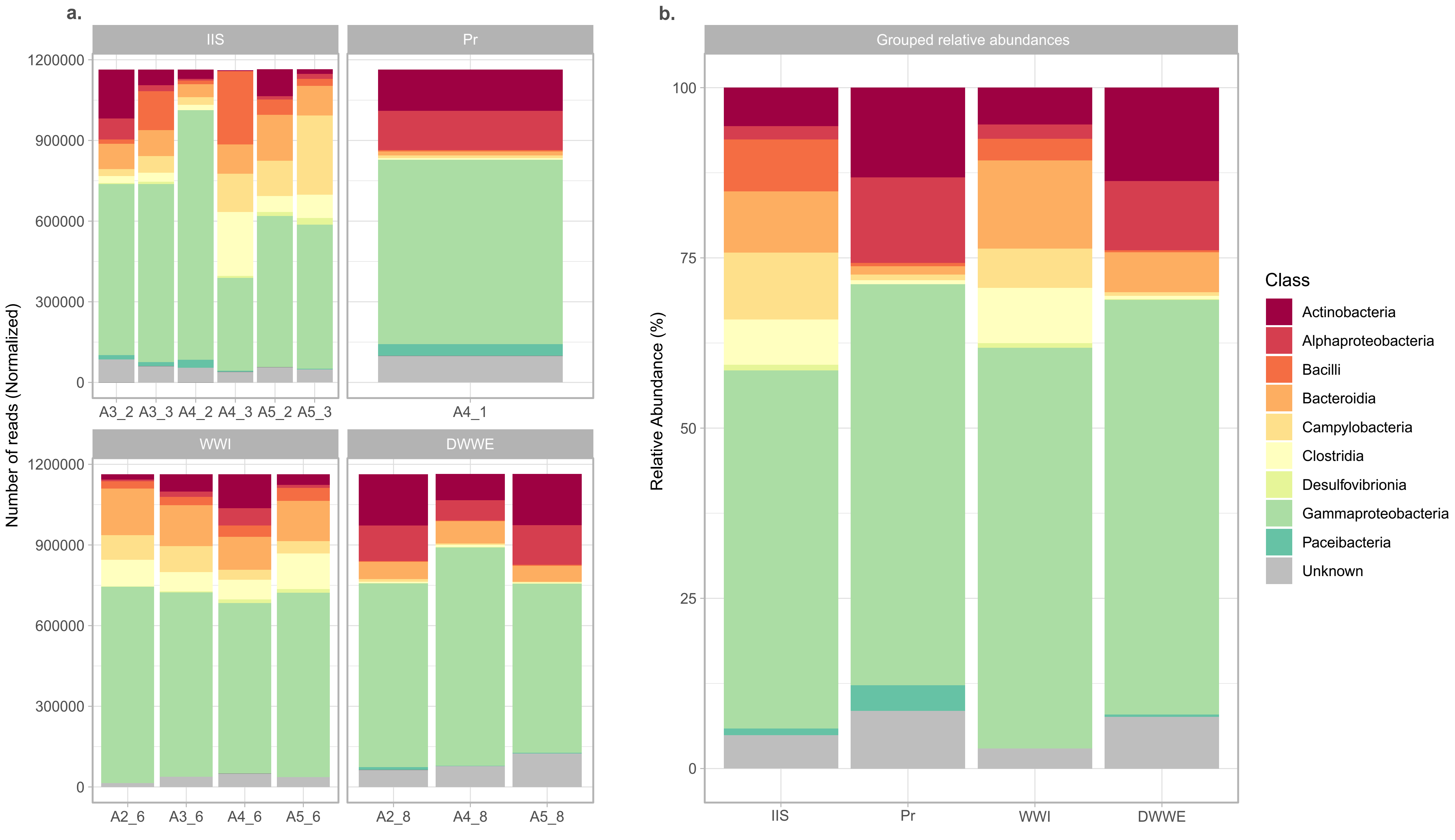

Figure S1.Diversity community profiles bar plots of the top 10 most abundant Classes. The bar plots are showing a. the normalised (median sequencing depth) number of reads for each sample in each sampling site b. and the averaged relative abundance (% of reads) for each sampling site.The labels on the X axis on the left panel denote the unique ID of each sample and on the right panel the ID of each sampling site.

Table S6.1-6.7: Source data for Figure 2. Percentage of antibiotic resistant bacteria grouped by antibiotic, site and sampling event.

Table S6.1: Pr

July 2018	September 2018	November 2018	January 2019	March 2019	May 2019
0.01	0.01	2.5		19.99341	14.02778
75.91666	33.7987	11.54762	0.9803922	44.52381	
20.58824	0.01	9.375		64.32806	2.152778
30	137.0779	2.261905	0.4901961	325.7143	3.26087
	0.01	0.01		0.7246377	0.01
0.01	0.01	0.01	0.01	2.142857	0.01
0.01	0.01	0.01		0.01	0.01
0.01	0.01	1.547619	0.01	0.01	0

Table S6.2: IIS

July 2018	September 2018	November 2018	January 2019	March 2019	May 2019
3.738985	29.15964	3.281462	0.384541	1.556244	3.314884
15.03175	9.747328	1.488395	0.755555		16.05171
21.48999		0.986421	0	2.300072	0.650508
211.1111	3.563986	20.58014	7.807092	5.507812	5.230975
9.492064	2.079853	6.557305	2.322222		1.822598
3.962923		4.662608	4.388681	7.225087	3.361791
0.837879	0.103388	0.223339	0.063704	2.740226	1.59954
127.2222	0.49326	2.359708	4.740741		0.661655
2.136256		2.163225	2.09108	7.936054	0.850746
6.312237	22.38454	48.29494	16.89837	46.14805	31.78025
3.888889	7.619606	117.9353	69.51667	0.01	22.73251
3.54851	0.01	50.02934	9.374063	106.8137	14.10448

Table S6.3: C1

July 2018	September 2018	November 2018	January 2019	March 2019	May 2019
1.877624	10.15885	3.282051	0.2678571	1.691919	5.736842
9.950419	560.9091	0.7919255	0.9854369	4.404762	12.29286
		1.186012	0	10.68966	2.774823
5.617	1.755132	16.01282	21.42857	8.106061	2.324561
1.449275	416.9091	1.545807	10.91262	40.11905	2.938424
		4.434524	8.145454	8.965517	8.37175
0.01	0.2311828	0.974359	0.6357143	3.328283	0.7982456
1.784897	125.6364	1.195652	1.563592		1.971579
		0.5372024	0.1481818	8.448276	0.7978724
0.01	2.888808	7.512821	0.6357143	15.88384	2.745614
1.300915	478.0909	6.886646	0.01	65.35714	21.23592
		11.13095	12.43636	24.13793	3.93617

Table S6.4: WWI

July 2018	September 2018	November 2018	January 2019	March 2019	May 2019
0.53529	3.064935	1.17352	2.103175	3.268495	1.553361
1.41176	3.428571	1.741776	2.202381	1.75287	1.884874
3	0.3295617	1.056743	1.884921	2.433036	0.9428571
	8.206169	15.02467	10.07936	6.246811	9.243697

Table S6.5: WWE

July 2018	September 2018	November 2018	January 2019	March 2019	May 2019
0.01	73.31895	0.01	0.01	5.787181	27.34625
0.01	2.326215	0.01	0.01	1.409134	1.183169
0.01	0.01	0.01	0.01	7.08067	0.01
0.01	0.22149	0.01	0.01	0.344868	0.01

Table S6.6: DWWE

July 2018	September 2018	November 2018	January 2019	March 2019	May 2019
26.95063	55.28104	5.001655	1.178788	18.58504	5.497191
3.846154		14.46488	0.462963	8.205646	13.65823
68.12461	3.224871	3.407084	5.318182	19.53079	3.722612
2.307692		4.531773	0.625	24.96416	0.6021429
4.812834	0.01	0.01	0.01	2.992669	0.01
1.726923		0.01	0.162037	6.785714	2.528571
0.01	1.238371	1.051093	0.03333333	1.188416	0.01
0.01		15.48495	0.01	9.67742	4.551948

Table S6.7: C2

July 2018	September 2018	November 2018	January 2019	March 2019	May 2019
57.04832	19.91694	2.120536	3.4375		15.22669
43.14706		1.825	1.220731	17.18615	299.0132
98.05672	2.347869	2.040179	32.1875	5.546154	6.283525
111.8137		4.675	0.7545688	213.9827	0.3506329
7.857143	0.2338389	0.01	0.01	2.271795	0.01
4.502941		0.225	0.7574244	1.177419	0.1453165
3.644958	4.45242	0.01	0.01	2.528205	0.01
1.535686		0.01	0.01	20.23809	0.7531645

Table S7.1-7.4: Source data for Figure 3. Percentage of antibiotic resistant bacteria grouped by antibiotic, matrix and site.

Table S7.1: Carbapenems

Aqueous	Jul-18	Sep-18	Nov-18	Jan-19	Mar-19	May-19
Pr	0.01	0.01	2.5		19.99341	14.02778
IIS	3.738985	29.15964	3.281462	0.384541	1.556244	3.314884
C1	1.877624	10.15885	3.282051	0.2678571	1.691919	5.736842
WWI	0.53529	3.064935	1.17352	2.103175	3.268495	1.553361
WWE	0.01	73.31895	0.01	0.01	5.787181	27.34625
DWWE	26.95063	55.28104	5.001655	1.178788	18.58504	5.497191
C2	57.04832	19.91694	2.120536	3.4375		15.22669
**Sediment**						
Pr	75.91666	33.7987	11.54762	0.9803922	44.52381	
IIS	15.03175	9.747328	1.488395	0.755555		16.05171
C1	9.950419	560.9091	0.7919255	0.9854369	4.404762	12.29286
WWI						
WWE						
DWWE	3.846154		14.46488	0.462963	8.205646	13.65823
C2	43.14706		1.825	1.220731	17.18615	299.0132
**Biofilm**						
Pr						
IIS	21.48999		0.986421	0	2.300072	0.650508
C1			1.186012	0	10.68966	2.774823
WWI						
WWE						
DWWE						
C2						

Table S7.2: Colistin

Aqueous	Jul-18	Sep-18	Nov-18	Jan-19	Mar-19	May-19
Pr	20.58824	0.01	9.375		64.32806	2.152778
IIS	211.1111	3.563986	20.58014	7.807092	5.507812	5.230975
C1	5.617	1.755132	16.01282	21.42857	8.106061	2.324561
WWI	1.41176	3.428571	1.741776	2.202381	1.75287	1.884874
WWE	0.01	2.326215	0.01	0.01	1.409134	1.183169
DWWE	68.12461	3.224871	3.407084	5.318182	19.53079	3.722612
C2	98.05672	2.347869	2.040179	32.1875	5.546154	6.283525
**Sediment**						
Pr	30	137.0779	2.261905	0.4901961	325.7143	3.26087
IIS	9.492064	2.079853	6.557305	2.322222		1.822598
C1	1.449275	416.9091	1.545807	10.91262	40.11905	2.938424
WWI						
WWE						
DWWE	2.307692		4.531773	0.625	24.96416	0.6021429
C2	111.8137		4.675	0.7545688	213.9827	0.3506329
**Biofilm**						
Pr						
IIS	3.962923		4.662608	4.388681	7.225087	3.361791
C1			4.434524	8.145454	8.965517	8.37175
WWI						
WWE						
DWWE						
C2						

Table S7.3: Gentamicin

Aqueous	Jul-18	Sep-18	Nov-18	Jan-19	Mar-19	May-19
Pr		0.01	0.01		0.7246377	0.01
IIS	0.837879	0.103388	0.223339	0.063704	2.740226	1.59954
C1	0.01	0.2311828	0.974359	0.6357143	3.328283	0.7982456
WWI	3	0.3295617	1.056743	1.884921	2.433036	0.9428571
WWE	0.01	0.01	0.01	0.01	7.08067	0.01
DWWE	4.812834	0.01	0.01	0.01	2.992669	0.01
C2	7.857143	0.2338389	0.01	0.01	2.271795	0.01
**Sediment**						
Pr	0.01	0.01	0.01	0.01	2.142857	0.01
IIS	127.2222	0.49326	2.359708	4.740741		0.661655
C1	1.784897	125.6364	1.195652	1.563592		1.971579
WWI						
WWE						
DWWE	1.726923		0.01	0.162037	6.785714	2.528571
C2	4.502941		0.225	0.7574244	1.177419	0.1453165
**Biofilm**						
Pr						
IIS	2.136256		2.163225	2.09108	7.936054	0.850746
C1			0.5372024	0.1481818	8.448276	0.7978724
WWI						
WWE						
DWWE						
C2	0					

Table S7.4: Sulfamethoxazole

Aqueous	Jul-18	Sep-18	Nov-18	Jan-19	Mar-19	May-19
Pr	0.01	0.01	0.01		0.01	0.01
IIS	6.312237	22.38454	48.29494	16.89837	46.14805	31.78025
C1	0.01	2.888808	7.512821	0.6357143	15.88384	2.745614
WWI		8.206169	15.02467	10.07936	6.246811	9.243697
WWE	0.01	0.22149	0.01	0.01	0.344868	0.01
DWWE	0.01	1.238371	1.051093	0.03333333	1.188416	0.01
C2	3.644958	4.45242	0.01	0.01	2.528205	0.01
**Sediment**						
Pr	0.01	0.01	1.547619	0.01	0.01	0
IIS	3.888889	7.619606	117.9353	69.51667	0.01	22.73251
C1	1.300915	478.0909	6.886646	0.01	65.35714	21.23592
WWI						
WWE						
DWWE	0.01		15.48495	0.01	9.67742	4.551948
C2	1.535686		0.01	0.01	20.23809	0.7531645
**Biofilm**						
Pr						
IIS	3.54851	0.01	50.02934	9.374063		
C1			11.13095	12.43636		
WWI						
WWE						
DWWE						
C2	0					

Table S8: Source data for Figure 4A.
Beta diversity values used to generate the PCoA plot.

	A2_6	A2_8	A3_2	A3_3	A3_6	A4_1	A4_2	A4_3	A4_6	A4_8	A5_2	A5_3	A5_6	A5_8
**A2_6**	0	0.874311127	0.809342442441834	0.7254037667	0.2282921961	0.9697780304	0.8712894436	0.848737	0.475645	0.842320	0.605539	0.701668	0.445403	0.899313
**A2_8**	0.874311127	0	0.388360084286083	0.6188142703	0.7719372678	0.8178909023	0.795572594	0.925731	0.687591	0.490717	0.796128	0.814395	0.883613	0.474747
**A3_2**	0.809342442	0.388360084	0	0.4201377432	0.7159116662	0.770867553	0.6461655662	0.848583	0.626603	0.544859	0.699498	0.733558	0.826435	0.562788
**A3_3**	0.725403766	0.618814270	0.420137743189406	0	0.6591576304	0.8094717391	0.6669701812	0.757350	0.669964	0.647978	0.630296	0.642217	0.774175	0.741099
**A3_6**	0.228292196	0.771937267	0.71591166621867	0.6591576304	0	0.9520932335	0.8489051805	0.833193	0.363914	0.762174	0.553764	0.651147	0.412993	0.823691
**A4_1**	0.969778030	0.817890902	0.770867553015914	0.8094717391	0.9520932335	0	0.7639900722	0.954345	0.932159	0.823107	0.933194	0.937313	0.968772	0.819379
**A4_2**	0.871289443	0.795572594	0.646165566167675	0.6669701812	0.8489051805	0.7639900722	0	0.875483	0.827362	0.797006	0.783244	0.808327	0.875125	0.859725
**A4_3**	0.848737561	0.925731586	0.848583250431694	0.7573506884	0.83319382	0.9543450076	0.875483054	0	0.834973	0.910045	0.739796	0.610535	0.828295	0.946427
**A4_6**	0.475645602	0.687591128	0.626603524816976	0.6699644828	0.3639142713	0.9321590618	0.8273626736	0.834973	0	0.655236	0.538037	0.633158	0.382658	0.734935
**A4_8**	0.842320059	0.490717120	0.544859005254961	0.6479784482	0.7621746118	0.8231078343	0.7970066397	0.910045	0.655236	0	0.730246	0.763197	0.828503	0.416586
**A5_2**	0.605539381	0.796128681	0.699498932093502	0.6302968264	0.5537642124	0.9331948506	0.7832448863	0.739796	0.538037	0.730246	0	0.437150	0.531220	0.804307
**A5_3**	0.701668093	0.814395173	0.73355843985331	0.6422177661	0.6511470042	0.9373135804	0.8083270061	0.610535	0.633158	0.763197	0.437150	0	0.634539	0.817723
**A5_6**	0.445403332	0.883613513	0.826435696464817	0.7741752462	0.4129935651	0.9687727111	0.8751250099	0.828295	0.382658	0.828503	0.531220	0.634539	0	0.882741
**A5_8**	0.899313945	0.474747025	0.562788776961383	0.7410991242	0.8236916578	0.8193796249	0.8597257485	0.946427	0.734935	0.416586	0.804307	0.817723	0.882741	0

Table S9.1-9.5: Source data for Figure 5. Average number of ARGs detected in each isolate obtained at each sample site and matrix.

Table S9.1: Carbapenems

	Aqueous
**Pr**	0 0
**IIS**	0 4 3 5 3 5 1 0 7 7
**C1**	2 0 7 0
**WWI**	5 1 2 3 3 4 6 3 5 1 5 5 5 5 2 7
**WWE**	1 2
**DWWE**	0 1
**C2**	2

	Biofilm
**Pr**	
**IIS**	5 4 2 2 2 2 2 0 0 2 0 0 1
**C1**	2 1 2
**WWI**	
**WWE**	
**DWWE**	
**C2**	

	Sediment
**Pr**	
**IIS**	0 1 1 0 3 1 2 2 2 0
**C1**	5 0 1 0 0
**WWI**	
**WWE**	
**DWWE**	0
**C2**	2 0

Table S9.2: Peptides

Aqueous

**Pr**	1	0														
**IIS**	0	0	0	0	0	2	3	0	0	2	2					
**C1**	0	1	2	1												
**WWI**	0	2	0	0	0	0	0	0	0	2	0	0	0	0	2	2
**WWE**	0	0														
**DWWE**	0	0														
**C2**	0															

Biofilm

**Pr**													
**IIS**	1	0	1	0	0	0	0	1	1	2	0	1	3
**C1**	0	0	0										
**WWI**													
**WWE**													
**DWWE**													
**C2**													

Sediment

**Pr**										
**IIS**	1	0	0	0	0	0	2	2	0	0
**C1**	0	1	0	1	1					
**WWI**										
**WWE**										
**DWWE**	1									
**C2**	0	0								

Table S9.3: Aminoglycosides

Aqueous

**Pr**	0	0														
**IIS**	0	4	3	5	3	5	1	0	7	7						
**C1**	2	0	7	0												
**WWI**	5	1	2	3	3	4	6	3	5	1	5	5	5	5	2	7
**WWE**	1	2														
**DWWE**	0	1														
**C2**	2															

Biofilm

**Pr**													
**IIS**	5	4	2	2	2	2	2	0	0	2	0	0	1
**C1**	2	1	2										
**WWI**													
**WWE**													
**DWWE**													
**C2**													

Sediment

**Pr**										
**IIS**	0	1	1	0	3	1	2	2	2	0
**C1**	5	0	1	0	0					
**WWI**										
**WWE**										
**DWWE**	0									
**C2**	2	0								

Table S9.4: Sulfonamides-trimethoprim

	Aqueous
**Pr**	0 0
**IIS**	0 2 2 2 2 1 2 0 0 4 4
**C1**	1 0 4
**WWI**	1 1 0 2 2 2 3 1 2 0 1 1 1 2 1 4
**WWE**	0 0
**DWWE**	0 0
**C2**	0

	Biofilm
**Pr**	
**IIS**	3 1 0 2 2 2 2 0 0 1 2 0 2
**C1**	0 0 2
**WWI**	
**WWE**	
**DWWE**	
**C2**	

	Sediment
**Pr**	
**IIS**	0 0 2 0 2 1 2 3 2 0
**C1**	1 0 1 0 0
**WWI**	
**WWE**	
**DWWE**	0
**C2**	0 0

Table S9.5: Multidrug

Aqueous

**Pr**	10	3	1	12	12																					
**IIS**	3	10	10	16	10	30	12	0	35	17	7	3	1	9	31	12	12	61	4	9	11	55	35	12	10	8
**C1**	3	24	11	25	13	41	12	21																		
**WWI**	6	5	5	24	44	5	5	3	6	7	24	24	29	13	13	42										
**WWE**	12	12	24	12	4	9																				
**DWWE**	16	14	14	13																						
**C2**	0	11	9																							

Biofilm

**Pr**																					
**IIS**	3	3	3	3	3	3	3	3	1	21	23	17	12	8	3	7	24	12	12	11	24
**C1**	4	5	0	8	13	12															
**WWI**																					
**WWE**																					
**DWWE**																					
**C2**																					

Sediment

**Pr**	1	1	7	12	14													
**IIS**	3	2	3	4	3	25	10	3	6	24	13	18	23	18	10	2	10	8
**C1**	4	3	3	3	8	10	10											
**WWI**																		
**WWE**																		
**DWWE**	3	14	13															
**C2**	1	1	12															

Table S10.1-10.5: Source data for Figure 6. Number of ARGs detected at each site in the metagenomes.

Table S10.1: Carbapenems

	July	September	November	January
**Pr**			0	
**IIS**		0	0	0
**WWI**		1		
**DWWE**	0		0	0

Table S10.2: Peptides

	July	September	November	January
**Pr**			0	
**IIS**		0	0	3
**WWI**	3	1	3	4
**DWWE**	0		0	0

Table S10.3: Aminoglycosides

	July	September	November	January
**Pr**			0	
**IIS**		4	12	19
**WWI**	30	25	28	31
**DWWE**	1		2	0

Table S10.4: Sulfonamides-trimethoprim

	July	September	November	January
**Pr**			0	
**IIS**		7	6	14
**WWI**	8	7	8	9
**DWWE**	1		1	1

Table S10.5: Multidrug

	July	September	November	January
**Pr**			0	
**IIS**		12	16	38
**WWI**	41	44	50	72
**DWWE**	11		6	8

Supplementary Material Legends

Supplementary Text S1.docx: DNA extraction protocol adapted from Crouse and Amorese (1987).

DNA extraction protocol that was used for single colonies.

Supplementary Table S1.xlsl: Metadata for whole community genomics.

Sequencing read information and metadata for metagenome samples from each site.

Supplementary Table S2.xlsl : Metadata for single colony genomics.

Identification of each isolate at each site, and sequencing read information.

Supplementary Text S2.docx : R script session output information.

Session information from the script used to detect antibiotic resistance genes.

Supplementary Table S3.xlsl : Operational Taxonomic Units (OTUs) tables from the metagenomic taxonomic profiling.

Operational Taxonomic Units (OTUs) tables before and after the filtering of low abundance species from the metagenomic taxonomic profiling.

Supplementary Figure S1.pdf: Supplementary Figure 1.

Diversity community profiles bar plots of the top 10 most abundant Classes. The bar plots are showing **a.** the normalised (median sequencing depth) number of reads for each sample in each sampling site **b.** and the averaged relative abundance (% of reads) for each sampling site.The labels on the X axis on the left panel denote the unique ID of each sample and on the right panel the ID of each sampling site.

Supplementary Table S4.xlsl: CARD output data for antibiotic resistance genes detected in the metagenomic samples used in this study.

Numbers in the table indicate the percentage of the read that matches a reference sequence of the corresponding gene. Genes were only counted as being present in a particular isolate if the percentage match was ≥80%. A dot indicates that the gene was not present in a particular sample.

Supplementary Table S5.xlsl: CARD output data for antibiotic resistance genes detected in single colony isolates obtained in this study.

Numbers in the table indicate the percentage of the read that matches a reference sequence of the corresponding gene. Genes were only counted as being present in a particular isolate if the percentage match was ≥80%. A dot indicates that the gene was not present in a particular isolate. Core genes can be identified by determining which genes are present in >90% of the isolates of a particular genus for genera that contained >10 isolates.

Supplementary Table S6.xlsl: Source data for Figure 2.

Percentage of antibiotic resistant bacteria grouped by antibiotic, site and sampling event.

Supplementary Table S7.xlsl: Source data for Figure 3.

Percentage of antibiotic resistant bacteria grouped by antibiotic, matrix and site.

Supplementary Table S8.xlsl: Source data for Figure 4A.

Beta diversity values used to generate the PCoA plot.

Supplementary Table S9.xlsl: Source data for Figure 5.

Average number of ARGs detected in each isolate obtained at each sample site and matrix.

Supplementary Table S10.xlsl: Source data for Figure 6.

Number of ARGs detected at each site in the metagenomes.
